# Supplementary material for: Endothelial Jagged1 Antagonizes Dll4/Notch Signaling in Decidual Angiogenesis during Early Mouse Pregnancy
Source: Int J Mol Sci. 2020 Sep 5;21(18):6477. doi: 10.3390/ijms21186477 (PMC7554752; doi:10.3390/ijms21186477)
Supplement: Supplementary file 1 [file ijms-21-06477-s001.pdf]

Supplementary Materials

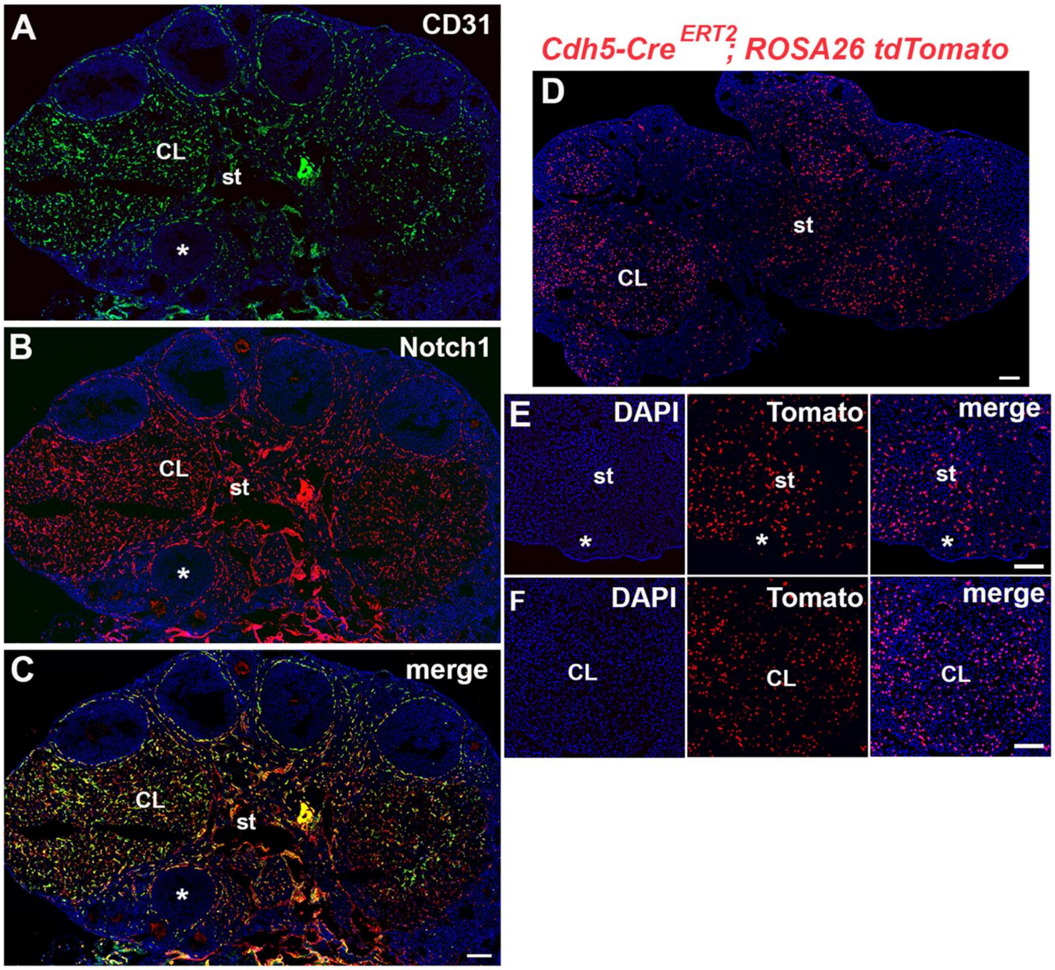

Supplementary Figure S1. Tamoxifen induces Cre recombination in ECs in *Cdh5-Cre*^{ERT2};*Jag1*$^{flox/flox}$ ovaries. (A-C) Representative image of an ovary at E7.5 double stained with EC marker CD31 and Notch1. Notch1 is expressed in CD31^{+} ECs in the corpus luteum (CL), stroma (st) and the theca layer around ovarian follicles (asterisk in ovarian follicle). (D) Representative images of an ovary at E7.5 from the *Cdh5-Cre*^{ERT2}; *ROSA26 tdTomato* reporter strain post administration of tamoxifen and progesterone at E4.5. tdTomato, indicating Cre expression, is detected in the vasculature of the ovary, including the corpus luteum (CL), stroma (st) and around the theca (*). Scale bars in C-F = 100 µm.

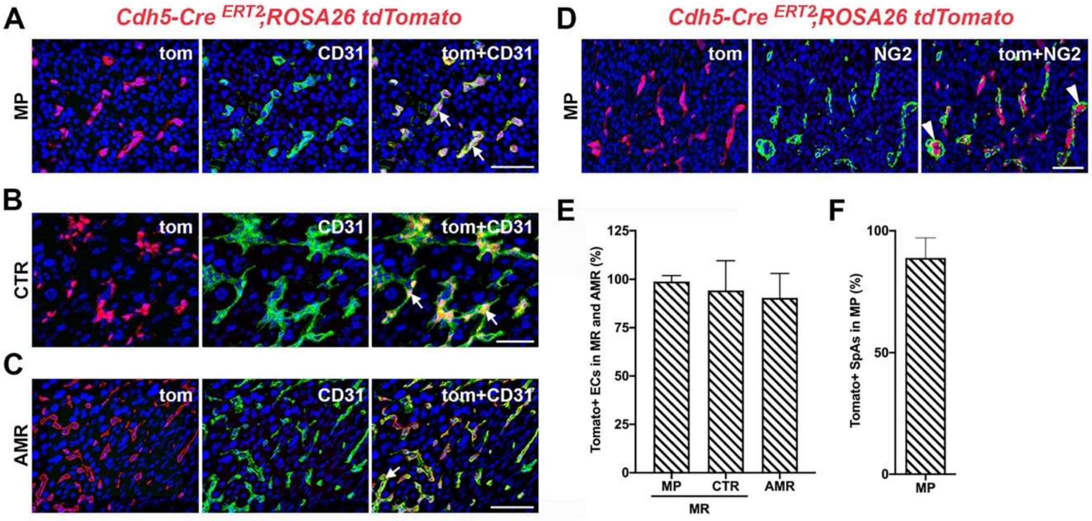

Supplementary Figure S2. *Cdh5-Cre*^{ERT2} recombinase is expressed in decidual spiral arteries and capillaries. Sections from *Cdh5-Cre*^{ERT2}; *ROSA26 tdTomato* pregnancies were stained with pericyte marker, NG2, and EC marker, CD31, to identify decidual vessels expressing *Cdh5-Cre*^{ERT2}. Tomato^{+} nuclei indicate expression of *Cdh5-Cre*^{ERT2}. (A) In the MP, SpAs have NG2^{+} pericytes closely associated with cells expressing *Cdh5-Cre*^{ERT2} (white arrowheads). (B) In the CTR, *Cdh5-Cre*^{ERT2} and CD31 are co-expressed in capillary ECs (white arrows). CTR = central region; MP = mesometrial pole; SpAs = spiral arteries; tom = tomato. Scale bars = 100 μm.

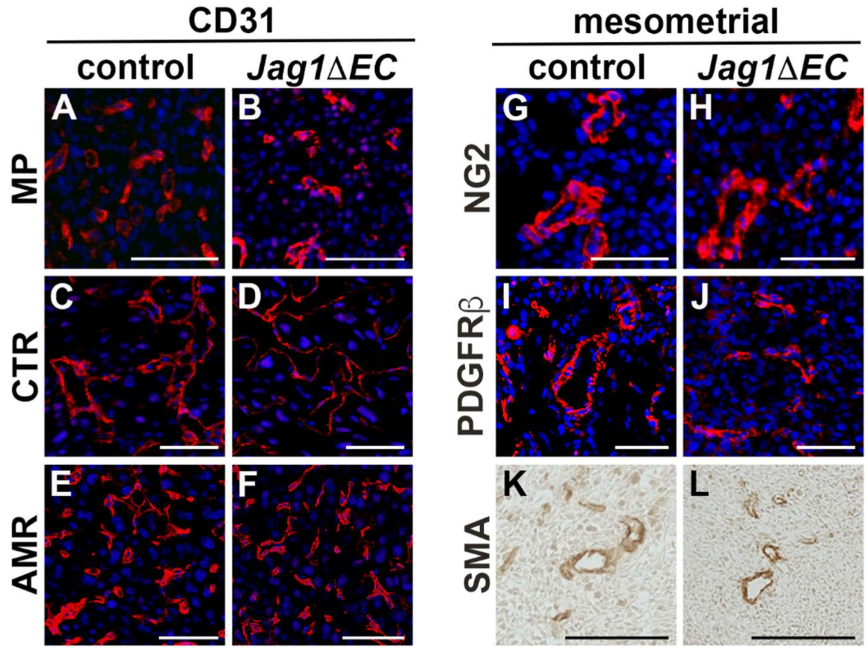

Supplementary Figure S3. Representative images of vasculature in *Cdh5-Cre*^{ERT2} control and *Jag1Δ EC* implantation sites at E7.5. High magnification images of CD31^{+} ECs in three regions of the implantation site, the MP (A, B), CTR (C, D), and AMR (E, F) at E7.5 in *Cdh5-Cre*^{ERT2} control and *Jag1Δ EC* pregnancies. (B) High magnification images of spiral arteries immunostained for mural cell markers, NG2 (G, H), PDGFRβ (I, J) or SMA (K, L) in the MP. AMR= anti-mesometrial region; CTR = central region; MP = mesometrial pole. Scale bars in A -J = 50 μm, K-L = 25 μm.

Supplementary Table S1. Primary and secondary antibodies used in immunostaining.

Name	Host	Company	Catalog Number	Concentration
<u>**Primary antibodies**</u>				
**CD31**	Rat	BD Sciences	553370	1:500
**Dll4**	Goat	R&D Systems	AF1389	1:100
**Jagged1**	Goat	R&D Systems	AF599	1:100
**Notch1**	Goat	R&D Systems	AF1057	1:200
**Notch1 (D1E11) XP**	Rabbit	Cell Signaling	3608S	1:100
**Notch4 Rb2-2**	Rabbit	Dr. Kitajewski's laboratory		1:100
**PDGFR-ß**	Rat	Abcam	Ab91066	1:100
**NG2**	Rabbit	Millipore	AB1057	1:750
**Alpha SMA**	Goat	Novus Biologicals	NB300-978SS	1:500
**Ki67**	Rabbit	Abcam	ab15580	1:200
**VEGFR2**	Goat	R&D Systems	AF644	1:100
<u>**Secondary antibodies**</u>				
**Anti-goat-IgG Alexa-Fluor 488**	Donkey	Invitrogen	A32814	1:1000
**Anti-goat-IgG Alexa-Fluor 594**	Donkey	Life Technologies	A11058	1:1000
**Anti-rabbit-IgG Alexa-Fluor 488**	Goat	Life Technologies	A11034	1:1000
**Anti-rabbit-IgG Alexa-Fluor 594**	Donkey	Invitrogen	A21207	1:1000
**Anti-rat-IgG Alexa-Fluor 488**	Donkey	Life Technologies	A21208	1:1000
**Anti-rat-IgG Alexa-Fluor 594**	Donkey	Life Technologies	A21209	1:1000
**Biotinylated anti-Goat**	Rabbit	Vector	BA-5000	1:400
**Biotinylated anti-Rat**	Rabbit	Vector	BA-4001	1:200
